# Economic and Cardiometabolic Risk Factors Are Predictors of Lower Thyroid Stimulating Hormone (TSH) Levels in Hispanic/Latinx Adults with Euthyroidism—A Community-Based Study

**DOI:** 10.3390/ijerph19138142

**Published:** 2022-07-02

**Authors:** Sabrina Sales Martinez, Margaret Gutierrez, Ivan Delgado-Enciso, Jezabel Maisonet, Aydevis Jean Pierre, Adriana Campa, Laura Kallus, Janet Diaz Martinez

**Affiliations:** 1 Robert Stempel College of Public Health and Social Work, Florida International University, Miami, FL 33199, USA; mguti140@fiu.edu (M.G.); ivan_delgado_enciso@ucol.mx (I.D.-E.); campaa@fiu.edu (A.C.); 2 Faculty of Medicine and Cancerology State Institute, University of Colima, Colima State Health Services, Colima 28040, CP, Mexico; 3 Caridad Center, Boynton Beach, FL 33472, USA; jmaisonet@caridad.org (J.M.); ajeanpierre@caridad.org (A.J.P.); lkallus@caridad.org (L.K.); 4 Research Center in a Minority Institution (FIU-RCMI), Florida International University, Miami, FL 33199, USA

**Keywords:** thyroid-stimulating hormone, income, food insecurity, Hispanic, cardiometabolic risk factors

## Abstract

Thyroid hormone abnormalities are among the most common endocrine disorders comorbidly suffered alongside metabolic syndrome and type 2 diabetes mellitus (T2DM), and within the euthyroid range they may also impact other outcomes, such as mood disorders. This study aimed to observationally examine the relationship between TSH and social determinants of health and clinical measures in a euthyroid Hispanic/Latinx patient sample with a diagnosis of anxiety and/or depression disorders from a community health clinic. A needs assessment was completed using a random sample of 100 de-identified medical records of individuals who received free medical care, including mental health, at a community-based clinic. Those with low normal TSH (<2 mIU/L) compared with high normal TSH (≥2 mIU/L) had a greater odds of food insecurity (*p* = 0.016) and being at 100% of the federal poverty level (*p* = 0.015). The low normal TSH group had significantly higher fasting glucose (*p* = 0.046), hemoglobin A1c (*p* = 0.018), and total cholesterol (*p* = 0.034) compared with the high normal TSH group. In those with T2DM, individuals with low normal TSH had six-times greater odds of having high fasting glucose (*p* = 0.022) and high hemoglobin A1c (*p* = 0.029). These relationships warrant further study, to inform future public health policies and follow-up care for underserved and vulnerable communities.

## 1. Introduction

Globally, thyroid dysfunction may affect about 5–8% of the population [1]. According to the American Thyroid Association, it is estimated that a thyroid condition may arise in more than 12% of the U.S. population during their lifetime [2]. This prevalence also varies by age, sex, race/ethnicity, and region, depending on the dietary intake of iodine [3,4,5]. Health outcomes related to cardiovascular disease (CVD), type 2 diabetes mellitus (T2DM), bone health, kidney function, and mood disorders may also be impacted by abnormal thyroid function [6,7,8,9].

Thyroid-stimulating hormone (TSH) is secreted by the pituitary gland and is commonly measured as part of clinical screenings for thyroid function [10]. Additional measures of thyroid function include the thyroid hormones triiodothyronine (T3) and thyroxine (T4). Often in primary care settings, TSH may be the only measure available; however, it remains one the of most useful tools available when assessing thyroid function [11]. TSH controls the release of T3 and T4 thyroid hormones, making it a sensitive marker. TSH, therefore, fluctuates due to the circadian rhythm and responds logarithmically to small deviations in thyroid hormones [12]. TSH may be affected by medications, illness, malnutrition, age, seasonality, pregnancy, and other hormones [13]. Euthyroid patients have normal TSH levels within the range of 0.45–4.0 mIU/L, and T3 within the normal reference range. Overt hypothyroidism is defined as elevated TSH and decreased T4. Subclinical hypothyroidism occurs when TSH is elevated but the thyroid hormones T3 and T4 are within the normal range. Overt hyperthyroidism is considered to include low levels of TSH and increased T3 and/or T4. Subclinical hyperthyroidism is defined as decreased TSH only. Subclinical thyroid dysfunction and euthyroidism in those with CVD or risk factors for CVD remains an understudied area in the literature [14]. 

Thyroid hormone excess and low levels may provoke or worsen CVDs [14]. Thyroid dysfunction may be a risk factor for CVD, within the euthyroid range [15,16,17]. Increased cardiovascular risk may be seen with a low normal thyroid function. In diverse populations, low normal or high normal TSH has been associated with cardiometabolic risk factors, such as fasting glucose, high-density lipoprotein (HDL) cholesterol, hemoglobin A1c (HbA1c), carotid intima-media thickness, higher heart rate, atrial fibrillation in euthyroid individuals, and coronary heart disease [15,17,18,19,20]. 

Thyroid disorders may also be associated with depression and anxiety in adults, especially when untreated [21]. Both a surplus, and inadequate levels of, thyroid hormones may affect mood and cognition [22]. Additionally, thyroid and mental health disorders may also share symptoms, such as apathy, fatigue, cognitive impairments, and even psychosis [8,9]. Depression and anxiety are more common in those with CVD risk factors such as T2DM than in the general population [23,24].

Health disparities are present in thyroid conditions because of multiple systemic and patient factors [25]. The social and demographic traits of individuals, groups, and their communities have a great influence on the overall health of populations [26]. These social determinants of health or the conditions in which people are born into, grow, work, live, and age contribute to our societies’ health and health care inequities [27]. Physician volume (amount of procedures performed), hospital location, and treatment cost, amongst others, can disproportionately affect health outcomes in thyroid dysfunction [28,29,30]. Previous studies have shown that Black and Hispanic patients have lower diagnoses of thyroid disorders, higher complication and mortality rates, and increased hospital costs compared to white patients [28,31,32]. Racial and ethnic minorities are also more likely to be diagnosed with more advanced diseases, due to delayed presentation to a physician, and receive suboptimal treatment compared to non-Hispanic White patients [25].

Limited research has explored the associations between thyroid function within normal ranges and social determinants of health and clinical factors associated with cardiometabolic risk in underrepresented adults from underserved and vulnerable communities. These same communities have higher rates of multimorbidity [33], which may increase the complexity of care management [34,35]. This study aimed to examine the relationship between TSH and social determinants of health and clinical measures in a euthyroid Hispanic/Latinx patient sample with a diagnosis of depression and/or anxiety from a community health clinic that provides free care services. 

## 2. Materials and Methods

### 2.1. Study Design

We conducted a needs assessment that involved an observational cross-sectional review of 100 random medical records from those who received free medical care at a community-based clinic and who were referred for mental health services by their primary care physicians (PCPs) during 2019. This community clinic serves the uninsured, underserved, and those who live under the federal level of poverty from the local community. Data were de-identified to the study investigators. 

The objective of the needs assessment was to identify pertinent co-morbid health issues associated with depression and anxiety in patients who receive care at the community clinic, to inform decisions on how best to improve health and wellness for the patients, and to address comorbidities. All of the patient data included were from Hispanic/Latinx individuals and all had a diagnosis of a mental health disorder, including depressive disorders (43.1%), anxiety disorders (38.2%), and both depressive and anxiety disorders (16.7%). All patients included in the analysis were receiving medical care provided by their PCPs and physician specialists, according to their comorbidities. This study did not require IRB approval or informed consent, since the data were fully de-identified to investigators at both institutions. 

### 2.2. Measures

Data were abstracted on current depression and anxiety diagnoses, and the PRAPARE (protocol for responding to and assessing patient assets, risk and experiences) questionnaire was used, which collects data on social determinants of health that include domains of personal characteristics (demographics), family and income (household size, housing status, and neighborhood), money and resources (education, income, food security, utility security, medicine/healthcare security, transportation), and social and emotional health (social integration/isolation and stress) [36,37]. Data on diagnoses of comorbidities were also provided, along with biomedical laboratory information, to further characterize the comorbidities and cardiometabolic risk. Multimorbidity was defined as having the presence of two or more chronic conditions. All diagnoses were coded based on the International Classification of Disease, tenth revision (ICD-10).

### 2.3. Statistical Analyses

All the statistical analyses were conducted using SPSS software (SPSS Inc., Chicago, IL, USA) version 17.0. Continuous variables were represented as mean ± standard deviation or median with interquartile range (IQR), depending on whether continuous data were normally distributed. Annual income was further divided into categories: 1 = USD 0–13,200; 2 = USD 13,201–17,700; 3 = USD 17,701–24,000; and 4 = USD 24,001 or more. For comparisons of continuous data and percentiles, an independent *t*-test or Mann–Whitney U-test was used. Chi-squared test was used for comparisons of categorical variables. A univariate logistic regression analysis was used to identify predictors of the TSH lowest levels. Previous studies have suggested using higher normal TSH levels of >2 mIU/L, as this has been associated with cardiovascular risk factors and thyroid disorder [38,39]. Therefore, the cut-off point for high normal TSH was ≥2 mIU/L and low normal TSH was <2 mIU/L. Significant predictors were subsequently added to the multivariable model, and forward stepwise logistic regression identified the most parsimonious model. The probability used for the stepwise regression was set at 0.20 for entry of variables and 0.25 for removal, based on recommendations for multivariate analysis in previous studies. A sub-analysis conducted in patients with T2DM used a univariate logistic regression analysis to detect if a low TSH level was a predictor of having glucose levels above the median (≥170 mg/dL) or above the median hemoglobin A1c (>8.5%). 

## 3. Results

### 3.1. Demographics

The characteristics of the 100 abstracted patients are displayed in Table 1. Out of the total 100 patients, seven were not used for the analysis, due to missing TSH data. There were no significant differences by anxiety and/or depression diagnoses and TSH continuous levels (*p* = 0.392) or by TSH groups (*p* = 0.315) (data not shown). There were no significant differences between those in the low normal TSH and high normal TSH groups for age, gender, BMI, hypertension, diabetes, CVD, having three or more CVD risk factors, or the number of comorbidities. There was a trend towards significance for greater chronic kidney disease (CKD) presence in the high TSH group compared to low TSH (8.3% vs. 0.0%, *p* = 0.064). However, due to the small number of individuals with a diagnosis of CVD and CKD, the results may not be reliable. Those with low normal TSH compared with high normal TSH, had a higher proportion of food insecurity (43.5% vs. 18.8%, *p* = 0.009) and income that fell below the federal poverty level (86.4% vs. 63.4%, *p* = 0.013). 

### 3.2. Factors Associated with Low TSH (2 mIU/L)

Several factors, including demographics, diagnoses, risk factors for CVD, comorbidities, substance use, employment status, food insecurity, and income at 100% of the federal poverty level, were examined for their association with low normal TSH levels (Table 2). In the univariate models, food insecurity (OR 3.33, 95% CI 1.31–8.44, *p* = 0.011) and being within 100% of the federal poverty level (OR 4.68, 95% CI 1.51–14.48, *p* = 0.007) was positively associated with the risk of having low normal TSH. These relationships remained in the multivariate models for food security (OR 3.69, 95% CI 1.27–10.75, *p* = 0.016) and 100% federal poverty level (OR 4.03, 95% CI 1.30–12.45, *p* = 0.015). All other factors included in the models were not significantly associated with low TSH (see Table 3).

Variables related to economic status (annual income, total family income, annual income categories, and household family size) were examined for their relationships with TSH group (Low TSH vs. high TSH). Patients with low normal TSH levels had a low annual income (*p* = 0.042), total family income (*p* = 0.042), and annual income categories (*p* = 0.049). Household size was not associated with the TSH groups. 

### 3.3. Relationships between Cardiometabolic Risk Factors and TSH Group

Table 4 displays the relationships between cardiometabolic risk factors and TSH groups in all participants. Significantly higher mean values of fasting glucose, hemoglobin A1c, and total cholesterol (*p* = 0.05) were found in the low normal TSH group compared with the high normal TSH group. No significant differences by TSH group were seen with triglyceride levels. 

In a sub-analysis, as shown in Table 5, a greater proportion of patients diagnosed with T2DM with higher than median fasting glucose (≥170 mg/dL) were found in the low normal TSH group compared to the high normal TSH group (62.5% vs. 20.0%, *p* = 0.020). Those with a low normal TSH had a six-times greater odds of having high fasting glucose compared to those with high normal TSH (OR 6.66, 95% CI 1.31–33.69, *p* = 0.022). A greater percentage of individuals with higher than the median hemoglobin A1c (≥8.5%) were found in the low normal TSH group compared with the high normal TSH group (62.5% vs. 21.4%, *p* = 0.028). A greater odds of having high hemoglobin A1c was found in those with low normal TSH (OR 6.11, 95% CI 1.19–31.16, *p* = 0.029) compared to high normal TSH. Higher median total cholesterol was also found to be more prevalent in the low normal TSH group (61.1% vs. 25.0%, *p* = 0.039) compared to high normal TSH. This result also led to a greater odds of having higher cholesterol in those with T2DM and low normal TSH levels (OR = 4.71, 95% CI 1.07–20.62, *p* = 0.039). 

## 4. Discussion

In the present study, euthyroid Hispanic/Latinx adults who received mental health care at a free community clinic were found to have an independent association between TSH and factors associated with poverty and food insecurity. Additionally, a relationship between low-normal TSH levels, fasting glucose, and hemoglobin A1c was also demonstrated. We believe this study may be the first to document these relationships between TSH and economic and cardiometabolic factors in uninsured and underserved Hispanic/Latinx adults. 

Our study showed that socioeconomic variables such as income and food insecurity were associated with a lower normal level of TSH. In Spain, Palacios et al. reported a relationship between thyroid dysfunction, including subclinical dysfunction and socioeconomic status, in a large adult sample [40]. Low income and food insecurity may be more problematic in populations at higher risk for chronic conditions, as sacrifices may be made to afford medical expenses [41]. Patients receiving care at free clinics are reported to be low-income, minorities, and suffer from multimorbidity [42]. Additionally, lower socioeconomic status may also impact proper chronic disease management, and overall healthcare expenditures may be greater [43,44,45]. Food insecurity is defined as the inability to afford balanced meals and uncertain access to foods and the quantity of foods. It is estimated that about 10.5% of households in 2019 had limited access to enough food for an active and healthy life [46]. Patients from the community center sampled in this study reported being food insecure at a higher rate, at over 30%. Data from the National Health and Nutrition Examination Survey (NHANES) demonstrated that Hispanic/Latinx households are more likely to be food insecure than non-Hispanic White households and that food insecurity was associated with elevated cardiometabolic risk factors in low-income households [47]. 

There is controversy in the literature about the normal range of TSH, especially since deviations within the normal range may still be associated with negative health outcomes [48]. We found significant associations when we divided TSH into two groups, based on the cut-off point of low normal TSH as <2 mIU/L and high normal ≥ 2 mIU/L. Both low and high levels of TSH have been associated with cardiovascular events and negative metabolic factors [48]. The findings in this study revealed that lower normal TSH levels were associated with higher fasting glucose, hemoglobin A1C, and total cholesterol; all factors associated with CVD. Further exploration of relationships with a diagnosis of CVD was not possible in this study, due to a small diagnosis group available in the data, which reduces the reliability of the analyses. In several studies, a relationship between TSH and the diagnosis of metabolic syndrome, having metabolic risk factors associated with a higher risk of chronic disease such as CVD, was observed in euthyroid patients with normal ranges of TSH levels. This is in contrast to a study showing that women with TSH ≥2 mIU/L were more insulin resistant than women with <2 mIU/L [49].

Environmental factors, such as lifestyle factors and pollutants, may affect TSH and thyroid hormone levels [50]. The community clinic examined for this study serves a predominantly poor Hispanic/Latinx population. Poverty and food insecurity may limit dietary choices and variability in the diet, such as Vitamin D, therefore affecting circulating TSH levels [50]. Living in underserved and poor neighborhoods may also increase the risk of exposure to pollutants that may lead to health disparities [51,52]. A further investigation of these factors in this population may yield a greater insight into the relationships with TSH reported. 

Individuals with T2DM in this sample who had high fasting glucose (≥170 mg/dL) were more likely to have low TSH levels. TSH levels are associated with hyperglycemia and insulin resistance in euthyroid individuals [6]. The inequities experienced in underserved populations, and as experienced in this sample, are known to impact both biological and behavioral outcomes related to T2DM disease management [53]. The findings from this study differed from another study conducted with euthyroid adults who had T2DM, in which a positive association between TSH levels and cardiometabolic risk factors was demonstrated [54]. High fasting glucose has been associated with adverse events, such as all-cause mortality and cardiovascular events, in patients with multimorbidity [55]. As the population ages and the risk of both obesity and multimorbidity increases, understanding how thyroid hormone dysfunction can influence chronic disease status may help address the health disparities suffered in disadvantaged and vulnerable populations. Recently, scientists from multidisciplinary fields have identified areas of interest for further exploration, which included identifying subgroups of individuals with preexisting CVD or those at higher risk for CVD that may benefit from an intervention in the euthyroid state, and recognition of differences among demographic subgroups, including race/ethnicity and issues of health disparities [14]. 

A relationship between depression and TSH levels is documented in the scientific literature; however, much of this research shows this relationship with levels of TSH above or below the normal range [56,57,58]. In our study, the participants were diagnosed with anxiety and/or depression disorders and TSH was in the normal range. In a study conducted by Medici et al. [59], adults over 55 years old who had TSH levels in the lower normal range had higher depressive symptom scores, after adjustment for covariates that included sex and age. The same study demonstrated that even variation within the normal range may increase the risk for greater depressive symptoms over time. Additionally, subclinical hyperthyroidism has been associated with depressive symptoms, while accounting for other covariates such as age, sex, and socioeconomic and health-related factors [60].

Our study has some limitations. Our study included a small sample size and was cross-sectional, which did not allow for the establishment of directionality in these relationships. The small sample size also affected the reliability of running a regression analysis simultaneously, including covariates. Most of the participants were female, which may also have influenced the levels of TSH [61]. TSH was the only indicator of thyroid function in our study, and we were not able to assess thyroid function wholly through other measures. In addition, we were not able to account for the current medical treatment and goals the patients were receiving for their chronic conditions or comorbidities. A strength of our study was the inclusion of an underrepresented and underserved population with, or at high risk for, chronic diseases, as most of the studies conducted on TSH and health outcomes were conducted in White European populations [48]. We strongly believe that this study, despite its limitations, adds important information to the literature, which may be used to design and justify necessary future research in this field. Moreover, the relationships identified in this study are unique, as this population, who received free medical care, is more susceptible to the burden of multiple chronic diseases, which requires a more holistic treatment plan that takes into account their social determinants of health, in order to improve health equity. 

Interventions are needed in primary and community settings with an interdisciplinary team approach that also addresses social needs to properly treat multimorbidity, which may improve health outcomes if mood disorders such as depression are also addressed [62]. Clinical professionals should be aware of the relationships between TSH levels in the euthyroid range and risks for morbidity and mortality, particularly in populations who are underserved. Small differences in serum TSH may contribute to the variations in metabolic factors associated with CVD. Understanding how thyroid function can influence the metabolic factors that influence chronic disease risk among patients experiencing multimorbidity may help address the health disparities suffered in disadvantaged populations. These relationships warrant further study, to inform future public health policies and follow-up care for underserved and vulnerable communities. Screening in these communities can promote early diagnosis and care, to reduce health disparities and the cost of healthcare utilization. 

## 5. Conclusions

In euthyroid Hispanic/Latinx patients receiving free medical, social, and mental health care at a community clinic, small differences in serum TSH may have contributed to the variations in metabolic factors associated with glycemic control. The data presented in this marginalized population also showed a relationship between TSH levels and poverty and food security, indicating that social determinants of health may play a role in chronic disease progression, and that these factors should be examined in future longitudinal studies, to allow translating the findings into targeted interventions. 

## Figures and Tables

**Table 1:** Characteristics of the study patients by TSH Levels.

Covariate	All *n* = 100	TSH †		*p* Value
		Low *n* = 46	High *n* = 47	
Age, years ^§^	51.9 ± 11.8	52.0 ± 12.5	52.8 ± 11.2	0.736
Female ^¶^	82.8%	82.6%	83.0%	0.589
BMI, kg/m^2 §^	29.9 ± 5.9	29.8 ± 5.6	30.1 ± 6.4	0.788
Hypertension ^¶^	45.7%	45.7%	45.8%	0.575
Type 2 Diabetes Mellitus ^¶^	34.0%	37.0%	31.3%	0.357
CVD ^¶^	9.6%	13.0%	6.3%	0.222
CKD ^¶^	4.3%	0.0%	8.3%	0.064
3 or more CVD risk factors	52.1%	58.7%	45.8%	0.149
Number of Comorbidities ^§^	1.4 ± 1.0	1.4 ± 1.0	1.3 ± 1.1	0.714
Multimorbidity ^¶^	79.1%	81.8%	76.6%	0.392
Alcohol ^¶^	10.9%	8.3%	13.6%	0.457
Smoking ^¶^	25.9%	31.0%	20.0%	0.272
Unemployment ^¶^	41.6%	41.9%	41.3%	0.564
Food Insecurity ^¶^	30.9%	43.5%	18.8%	0.009 *
FPL 100% ^¶^	75.3%	86.4%	63.4%	0.013 *

†: thyroid-stimulating hormone; low TSH was defined as <2 mIU/L and high TSH as ≥2 mIU/L. ^§^: Student’s *t*-test (mean ± standard deviation); ^¶^: Chi-square test (%). *: Statistically significant (*p* < 0.05). BMI, body mass index; CVD, cardiovascular disease; CKD, chronic kidney disease; FPL, federal poverty level.

**Table 2:** Univariate and Multivariate Logistic Regression for Low TSH (<2 mIU/L) †.

Covariate		95% CI				95% CI		
	Unadjusted OR	Lower	Upper	*p* Value	Adjusted OR	Lower	Upper	*p* Value
Age, years	0.99	0.96	1.02	0.733	-	-	-	-
Female	1.02	0.35	3.01	0.962	-	-	-	-
BMI, kg/m^2^	0.98	0.91	1.05	0.655	-	-	-	-
Hypertension	0.99	0.44	2.23	0.986	-	-	-	-
Type 2 Diabetes Mellitus	1.29	0.54	3.03	0.364	-	-	-	-
CVD	2.25	0.52	9.59	0.273	-	-	-	-
CKD	2.15	0.50	9.20	0.301	-	-	-	-
3 or more CVD risk factors	1.67	0.74	3.80	0.213	-	-	-	-
Multimorbidity	1.37	0.49	3.81	0.541	-	-	-	-
Unemployment	1.02	0.44	2.37	0.958	-	-	-	-
Food insecurity	3.33	1.31	8.44	0.011 *	3.69	1.27	10.75	0.016 *
FPL 100%	4.68	1.51	14.48	0.007 *	4.03	1.30	12.45	0.015 *

† thyroid-stimulating hormone; low TSH was defined as <2 mIU/L and high TSH as ≥2 mIU/L. BMI, body mass index; CVD, cardiovascular disease; CKD, chronic kidney disease; FPL, federal poverty level. * Statistically significant (*p* < 0.05).

**Table 3:** Comparison of Economic Variables by TSH Group †.

	Low Normal TSH (*n* = 46)	High Normal TSH (*n* = 47)	*p* Value ^§^
	Median (IQR)	Median (IQR)	
Annual income, USD	15,060 (10,500)	19,200 (14,400)	0.042 *
Total Family Income, USD	1255 (875)	1600 (1200)	0.042 *
Annual income categories, USD	2 (2)	3 (2)	0.049 *
Household Family Members, *N*	4 (3)	4 (3)	0.938

† thyroid-stimulating hormone; low TSH was defined as <2 mIU/L and high TSH as ≥2 mi/L. § Mann–Whitney U-test. IQR: interquartile range. * Statistically significant (*p* < 0.05).

**Table 4:** Cardiometabolic Risk Factors by TSH Group (*n* = 93) †.

Metabolic Variables	Low Normal TSH (*n* = 46)		High Normal TSH (*n* = 47)		*p* Value ^§^
	Mean	SD	Mean	SD	
Fasting glucose, mg/dL	194.0	86.2	140.6	52.8	0.046 *
Hemoglobin A1c, %	9.6	2.8	7.5	1.5	0.018 *
Total cholesterol, mg/dL	200.5	32.8	171.5	39.7	0.034 *
Triglycerides, mg/dL	180.0	111.5	157.0	80.7	0.524

† thyroid-stimulating hormone; low TSH was defined as <2 mIU/L and high TSH as ≥2 mIU/L. ^§^ Student *t*-test. * Statistically significant (*p* < 0.05).

**Table 5:** TSH as a Predictor for Glucose and Lipid Control in Type 2 Diabetic Patients (*n* = 34) †^¶^.

		Fasting Glucose (mg/dL)				95% CI		
		≤169	≥170		Crude OR *	Lower	Upper	*p*
TSH	High	80.0%	20.0%	100%	Reference			
	Low	37.5%	62.5%	100%	6.66	1.31	33.69	0.022 *
		Hemoglobin A1c (%)						
		≤8.4	≥8.5					
TSH	High	78.6%	21.4%	100%	Reference			
	Low	37.5%	62.5%	100%	6.11	1.19	31.16	0.029 *
		Total cholesterol (mg/dL)						
		≤189	≥190					
TSH	High	75.0%	25.0%	100%	Reference			
	Low	38.9%	61.1%	100%	4.71	1.07	20.62	0.039 *

† thyroid-stimulating hormone; low TSH was defined as <2 mIU/L and high TSH as ≥2 mIU/L. ^¶^ Univariate logistic regression analysis. * Statistically significant (*p* < 0.05).

## Data Availability

Data can be requested from the corresponding author.
